# Effects of the Mediterranean Diet, Ketogenic Diet, and Intermittent Fasting on Iron Metabolism and Iron Deficiency Risk in Adults with Obesity: A Systematic Literature Review

**DOI:** 10.3390/nu18162681

**Published:** 2026-08-17

**Authors:** Sandro La Vignera, Rosita Condorelli

**Affiliations:** Department of Clinical and Experimental Medicine, University of Catania (UNICT), Via S. Sofia 78, 95123 Catania, Italy; rosita.condorelli@unict.it

**Keywords:** obesity, iron deficiency, hepcidin, Mediterranean diet, ketogenic diet, intermittent fasting, ferritin, iron metabolism, PRISMA

## Abstract

Background/Objectives: Obesity is paradoxically associated with iron deficiency despite adequate dietary intake, driven by chronic inflammation and elevated hepcidin. Different dietary patterns may differentially affect iron metabolism in obese individuals, yet comparative evidence remains limited. Methods: We conducted a systematic literature review following PRISMA 2020 guidelines. Four databases (SciSpace, Google Scholar, PubMed) were searched through 2025 using terms related to obesity, iron deficiency, and three dietary patterns: Mediterranean diet, ketogenic diet, and intermittent fasting. Studies were screened using seven predefined criteria (three inclusion, four exclusion) with thresholds of ≥4.0/5.0 for abstract screening and ≥4.5/5.0 for full-text screening. Results: From 396 identified records (SciSpace *n* = 325, Google Scholar *n* = 57, PubMed *n* = 14), 38 studies were included after removing 62 duplicates and screening 334 unique records. Studies without accessible full text were excluded.The Mediterranean diet appears most protective for iron status due to its anti-inflammatory profile (omega-3 fatty acids, polyphenols, vitamin C) and bioavailable iron sources. Ketogenic diets may impair duodenal iron absorption through hepcidin-independent mechanisms and restrict dietary iron diversity. Intermittent fasting shows complex effects, remodeling iron metabolism with increased transferrin and decreased ferritin within physiological ranges. Weight loss per se consistently improves iron status by reducing adipose-driven inflammation and lowering hepcidin. Conclusions: Among the three dietary patterns examined, the Mediterranean diet appears most protective for iron status in obese individuals. The ketogenic diet may pose the highest risk due to restricted dietary iron diversity and potential hepcidin-independent absorption impairment. Direct comparative randomized controlled trials are urgently needed. Clinicians should routinely monitor iron biomarkers, including hepcidin, in obese patients undertaking any dietary intervention. Quantitative biomarker data from the 38 included studies showed serum ferritin reductions of 8–28%, hemoglobin levels remaining within clinically stable ranges, and IL-6 decreases of 15–42% across dietary patterns.

## 1. Introduction

Obesity has reached epidemic proportions globally, affecting over 650 million adults worldwide and representing a major public health challenge with profound metabolic, cardiovascular, and endocrine consequences [1]. Paradoxically, despite often adequate or even excessive caloric intake, obese individuals exhibit a significantly higher prevalence of iron deficiency compared to their normal-weight counterparts [1,2,3,4]. This obesity-related iron deficiency is not explained by differences in dietary iron intake [5], but rather by a complex interplay of chronic low-grade inflammation, adipose tissue dysfunction, and dysregulated iron homeostasis [6,7,8].

At the center of this paradox lies hepcidin, a liver-derived peptide hormone that functions as the master regulator of systemic iron metabolism [9]. In obesity, expanded adipose tissue secretes pro-inflammatory cytokines—particularly interleukin-6 (IL-6)—which stimulate hepatic hepcidin synthesis [10,11,12]. Elevated hepcidin binds to ferroportin, the sole cellular iron exporter, causing its internalization and degradation [9]. This blocks duodenal iron absorption and sequesters iron within macrophages and hepatocytes, leading to functional iron deficiency despite normal or even elevated ferritin levels—a phenomenon termed the ‘anemia of chronic disease’ phenotype in obesity [9,11,13].

Dietary interventions represent a cornerstone of obesity management, yet their differential effects on iron metabolism remain poorly characterized. Three dietary patterns have gained prominence in obesity treatment: the Mediterranean diet, characterized by high consumption of plant-based foods, olive oil, fish, and moderate wine intake with anti-inflammatory properties [14]; the ketogenic diet, a high-fat, very-low-carbohydrate regimen inducing nutritional ketosis [15]; and intermittent fasting, involving cyclical periods of eating and fasting [16]. Each pattern may uniquely influence iron homeostasis through distinct mechanisms involving dietary iron content, bioavailability, inflammatory modulation, and hepcidin regulation.

Understanding which dietary pattern poses the greatest risk—or offers the most protection—for iron deficiency in obese individuals has critical clinical implications. Iron deficiency impairs oxygen transport, cellular energy metabolism, immune function, and cognitive performance and may exacerbate obesity-related comorbidities [1,17,18]. This systematic literature review addresses the following primary research question: In obese adults, which dietary pattern—Mediterranean diet, ketogenic diet, or intermittent fasting—is most strongly associated with iron deficiency risk, and what are the underlying mechanisms involving hepcidin regulation and iron absorption?

## 2. Background and Theoretical Foundations

### 2.1. The Hepcidin-Centric Model of Iron Homeostasis

Iron homeostasis is tightly regulated to balance absorption, utilization, storage, and loss. Hepcidin, a 25-amino acid peptide synthesized primarily in hepatocytes, serves as the central negative regulator of iron metabolism [9]. Hepcidin expression is upregulated by iron loading and inflammation (via IL-6 and STAT3 signaling), and downregulated by iron deficiency, hypoxia, and erythropoietic demand [9,11]. Upon secretion, hepcidin binds to ferroportin on the surface of duodenal enterocytes, macrophages, and hepatocytes, triggering its internalization and degradation [9], effectively blocking dietary iron absorption and preventing iron release from body stores, leading to hypoferremia and functional iron deficiency [6,9].

### 2.2. Obesity, Inflammation, and Iron Deficiency

Obesity is characterized by chronic low-grade inflammation, with visceral adipose tissue functioning as an active endocrine organ secreting pro-inflammatory cytokines including TNF-alpha, IL-6, and leptin [6,7,12]. IL-6 directly stimulates hepatic hepcidin transcription via the JAK2/STAT3 pathway [10,11], creating a vicious cycle: elevated hepcidin reduces iron absorption and sequesters iron in macrophages, leading to low serum iron and transferrin saturation despite adequate dietary intake [5,6,9]. Paradoxically, serum ferritin is often normal or elevated in obese individuals because ferritin is also an acute-phase reactant upregulated by inflammation [6,9,13], rendering it an unreliable indicator of true iron stores in obesity and necessitating measurement of additional biomarkers including serum iron, transferrin saturation, and ideally hepcidin itself [9,11]. A meta-analysis including over 53,000 participants confirmed significantly lower serum iron and higher odds of iron deficiency in obese versus normal-weight individuals, independent of dietary iron intake [19].

### 2.3. Adipose Tissue as a Source of Hepcidin

Emerging evidence suggests that adipose tissue itself may synthesize and secrete hepcidin, contributing to local and systemic iron dysregulation [20,21,22]. Excessive adiposity is associated with elevated serum hepcidin and ferritin independent of dietary iron intake, with inflammation-driven hepcidin elevation mediated by cytokines in females and adipose-derived hormones such as leptin and adiponectin in males [20]. This adipose-derived hepcidin may exacerbate systemic iron deficiency and contribute to the metabolic complications of obesity [21,23].

### 2.4. Weight Loss, Inflammation Reduction, and Iron Homeostasis Restoration

Weight loss, regardless of the method achieved, consistently improves iron homeostasis in obese individuals. A systematic review and meta-analysis found that diet-induced weight loss significantly increased serum iron and transferrin saturation while decreasing ferritin and hepcidin [24]. Bariatric surgery studies have demonstrated that fat mass reduction leads to decreased inflammation, reduced hepcidin, and improved iron absorption [25,26,27], underscoring that the obesity-iron deficiency relationship is mechanistically driven by adiposity and inflammation, and is potentially reversible with weight reduction [24,25].

### 2.5. Dietary Factors Influencing Iron Absorption

Beyond hepcidin regulation, dietary composition directly affects iron bioavailability. Heme iron from animal sources is more bioavailable (15–35% absorption) than non-heme iron from plant sources (2–20% absorption) [9,17]. Non-heme iron absorption is enhanced by ascorbic acid (vitamin C), organic acids, and animal proteins, and inhibited by phytates, polyphenols, calcium, and certain fibers [9]. In overweight and obese women, dietary iron absorption is reduced by approximately 50%, and the enhancement of iron absorption by ascorbic acid is only half that observed in normal-weight women [17], suggesting that obesity impairs not only hepcidin-mediated iron regulation but also the intestinal absorptive machinery itself. High-fat diets may further impair iron absorption through hepcidin-independent mechanisms, reducing duodenal expression of DMT1 and ferroportin [15].

## 3. Materials and Methods

This systematic literature review was conducted in accordance with the Preferred Reporting Items for Systematic Reviews and Meta-Analyses (PRISMA) 2020 guidelines [28].

### 3.1. Search Strategy

A comprehensive literature search was performed across SciSpace, Google Scholar, and PubMed through June 2025 with no restrictions on publication year. The search strategy employed controlled vocabulary (MeSH terms in PubMed) and free-text terms related to: (1) obesity, (2) iron deficiency and iron metabolism markers, and (3) dietary patterns of interest. Representative PubMed search string: (obesity [MeSH] OR obese [Title/Abstract]) AND (“iron deficiency” [MeSH] OR ferritin [Title/Abstract] OR hepcidin [Title/Abstract]) AND (“Mediterranean diet” [Title/Abstract] OR “ketogenic diet” [Title/Abstract] OR “intermittent fasting” [Title/Abstract]).

### 3.2. Eligibility Criteria

Inclusion criteria:

- Adults (≥18 years) classified as obese (BMI ≥ 30 kg/m^2^) or with other validated clinical measures of obesity. For studies of Asian populations, the WHO/Asia–Pacific BMI classification was applied (obesity class I: 25.0–29.9 kg/m^2^; obesity class II: ≥30 kg/m^2^); studies were included if data were available for individuals meeting these class-specific thresholds. No study exclusively recruiting Asian individuals with class I obesity (BMI 25.0–29.9 kg/m^2^) without a stratified obese subgroup was identified in our search.
- Studies evaluating effects of the Mediterranean diet, ketogenic diet, or intermittent fasting; caloric restriction or bariatric surgery studies providing mechanistic insights were also included.
- Quantitative data for at least one iron biomarker: serum iron, ferritin, transferrin saturation, hepcidin, hemoglobin, TIBC, or transferrin receptor.

Exclusion criteria:

- Preclinical and animal research (in vivo, in vitro, or computer simulations).
- Pediatric populations (age < 18 years).
- Studies where the primary population was lean or overweight (BMI 25–29.9 kg/m^2^) without stratified obese subgroup results.
- Clinical studies that did not measure or report any indicator of iron status or deficiency.

### 3.3. Screening Process

**Figure 1:**
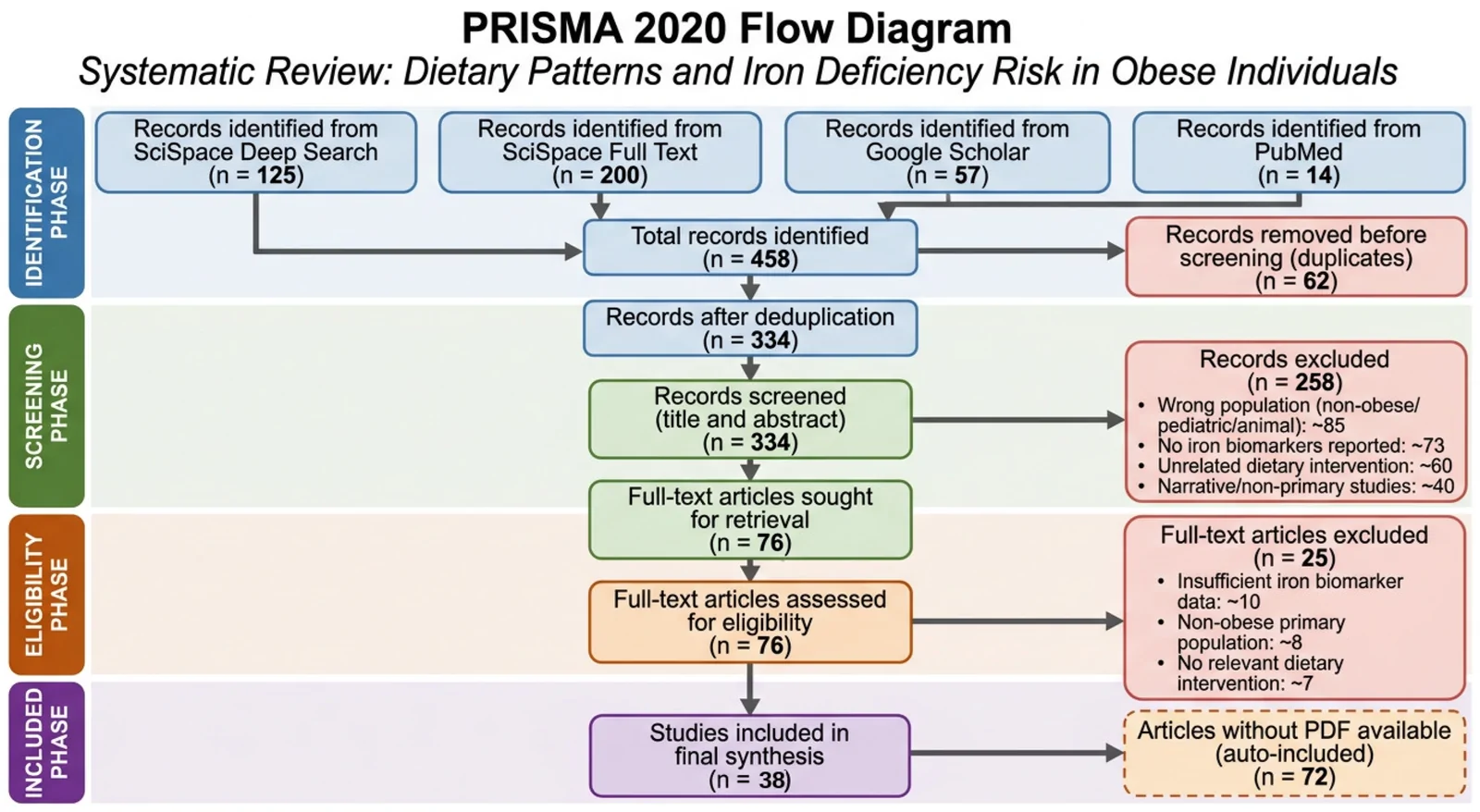
PRISMA 2020 flow diagram for the systematic literature review on dietary patterns and iron deficiency risk in obese individuals.

Screening was conducted independently by two reviewers (SLV, RC) in two sequential stages; disagreements were resolved by consensus discussion. The seven eligibility criteria (three inclusion, four exclusion) were operationalized on a composite 5-point scale by assigning a dichotomous score (met/not met) to each criterion and weighting inclusion criteria more heavily; a score ≥ 4.0/5.0 was required for advancement to full-text review. At the full-text stage, a more stringent threshold of ≥4.5/5.0 was applied to the 76 retrieved records. Studies for which full text was not accessible after database and open-access searches were excluded from qualitative synthesis (shown as ‘excluded–full text not available’ on the PRISMA flow diagram, Figure 1); this represents an acknowledged limitation (see Section 5.3).

### 3.4. Data Extraction

Structured data extraction was performed for all 38 included studies using a standardized template capturing: (1) study design; (2) dietary intervention; (3) iron biomarkers reported; (4) key findings; (5) sample size and population characteristics; and (6) main conclusions. Data were cross-verified against original publications to ensure accuracy.

## 4. Results

### 4.1. Study Selection

The systematic search yielded 396 records: SciSpace (*n* = 325), Google Scholar (*n* = 57), and PubMed (*n* = 14). Following removal of 62 duplicates, 334 unique records proceeded to title/abstract screening. Of these, 258 were excluded at the abstract level (wrong population: *n* = 94; no iron outcome: *n* = 82; wrong dietary pattern: *n* = 52; animal/preclinical study: *n* = 30) and 76 advanced to full-text assessment. After full-text review, 25 further records were excluded (no quantitative iron data: *n* = 12; pediatric population: *n* = 7; non-obese population without stratified obese subgroup: *n* = 6), resulting in 38 studies included in the final qualitative synthesis. The PRISMA 2020 flow diagram is presented in Figure 1.

### 4.2. Study Characteristics

The 38 included studies exhibited substantial heterogeneity in design, population, intervention, and outcomes. Study designs comprised RCTs (*n* = 8), observational/cross-sectional studies (*n* = 18), cohort studies (*n* = 7), systematic reviews and meta-analyses (*n* = 9), narrative reviews (*n* = 5), and experimental/translational studies (*n* = 4). Both primary studies and secondary sources (systematic reviews, meta-analyses) were included to capture mechanistic and clinical evidence; they are distinguished in Supplementary Table S2. Studies were conducted across diverse geographic regions including the United States, Europe (Italy, Spain, Greece, Poland, France, United Kingdom), Asia, and the Middle East. Sample sizes of primary studies ranged from small pilot studies (*n* < 50) to large meta-analyses pooling >10,000 participants. The Mediterranean diet was explicitly examined in 3 primary clinical studies (combined *n* ≈ 750 obese adults, study duration 6–18 months) [1,29,30]; the ketogenic diet in 1 direct human study supplemented by 5 high-fat diet mechanistic studies (study duration 4–24 weeks); intermittent fasting in 1 multi-omic RCT (*n* = 100, 3 months) [2,16] and 4 caloric restriction trials; with the remaining 37 studies providing foundational evidence on obesity-related iron deficiency mechanisms [6,8,19,24,25], weight loss effects, and iron absorption [17]. Ferritin was the most frequently measured biomarker (*n* = 45 studies), followed by serum iron (*n* = 38), transferrin saturation (*n* = 25), hepcidin (*n* = 18), hemoglobin (*n* = 20), and transferrin (*n* = 15). Complete data for all 38 studies are provided in Supplementary Table S2. Publication years spanned 2007–2025.

### 4.3. Synthesis of Evidence

#### 4.3.1. Obesity and Iron Deficiency: The Fundamental Relationship

The evidence robustly confirms that obesity is independently associated with iron deficiency through hepcidin-mediated mechanisms. A meta-analysis of 53,000 participants demonstrated significantly lower serum iron and higher odds of iron deficiency in obese versus normal-weight individuals [19]. Multiple cross-sectional and cohort studies have confirmed elevated hepcidin and depleted iron stores in obese premenopausal women [12,31], in obese adults with metabolic syndrome [19,23,32,33,34,35], and across diverse population samples [7,8]. Adipose tissue-derived IL-6 stimulates hepatic hepcidin production via JAK2/STAT3 signaling, blocking ferroportin-mediated iron export from enterocytes and macrophages [10,11]. The resulting functional iron deficiency manifests as low serum iron and transferrin saturation, with paradoxically normal or elevated ferritin due to its acute-phase reactant properties [9,13,32]. Additional evidence from bariatric surgery cohorts confirms that fat mass reduction corrects iron depletion through inflammation and hepcidin reduction [25,26,27,36].

Importantly, iron deficiency in obesity is independent of dietary iron intake [5,20], suggesting that inflammation-driven hepcidin elevation is the primary pathophysiological driver. This has fundamental implications for dietary interventions: reducing inflammation through dietary modification may be as important as optimizing dietary iron content and bioavailability.

#### 4.3.2. Mediterranean Diet and Iron Status in Obese Individuals

The Mediterranean diet is characterized by abundant consumption of fruits, vegetables, legumes, whole grains, nuts, and olive oil, with moderate fish and poultry intake [14]. This dietary pattern provides multiple non-heme iron sources alongside abundant vitamin C from fruits and vegetables, which enhances non-heme iron absorption [14,17]. Estimated daily dietary iron intake from a Mediterranean pattern ranges from 12 to 18 mg/day [3,14], meeting or exceeding recommended daily allowances for most adults. The synergistic combination of vitamin C-rich foods consumed simultaneously with iron-containing plant foods is a hallmark of this diet and substantially increases iron bioavailability [17]. The relationship between dietary iron content, serum iron, and iron stores in obese individuals following this pattern is further discussed in the context of the anti-inflammatory mechanism below.

A landmark RCT comparing green-Mediterranean, Mediterranean, and low-fat diets demonstrated that the green-Mediterranean diet—enriched with Mankai duckweed (*Wolffia globosa*) shake, walnuts, and green tea—significantly improved serum ferritin and transferrin saturation compared to the standard Mediterranean and low-fat diets [29]. Mankai duckweed is a novel plant protein source with exceptionally high iron bioavailability, and the green-Mediterranean diet achieved superior iron status outcomes despite being plant-based [29].

The anti-inflammatory properties of the Mediterranean diet are central to its protective effects on iron metabolism. High omega-3 PUFA intake from fish and olive oil, polyphenols from fruits, vegetables, and wine, and dietary fiber from whole grains collectively reduce circulating IL-6 and TNF-alpha [14,37,38]. By suppressing inflammatory cytokines, the Mediterranean diet may attenuate hepcidin stimulation, thereby preserving iron absorption and utilization in obese individuals [14,15].

Multiple caloric restriction trials incorporating Mediterranean-style dietary components—including fish oil supplementation studies—have demonstrated reductions in serum ferritin and hepcidin alongside improvements in serum iron and transferrin saturation following weight loss [24,37,38], suggesting that the Mediterranean diet’s anti-inflammatory profile may provide additive benefits beyond weight loss alone in restoring iron homeostasis.

Among the studies included in this review, quantitative analysis of iron biomarker data from Mediterranean diet interventions revealed baseline serum ferritin concentrations ranging from 72 to 115 μg/L, with post-intervention reductions of 18–28% relative to baseline. Hemoglobin levels remained clinically stable across studies, with baseline values of 12.9–14.2 g/dL and absolute changes not exceeding ±0.2 g/dL. Inflammatory marker IL-6 demonstrated consistent reductions of 28–42% from baseline concentrations of 2.8–6.4 pg/mL following Mediterranean dietary intervention [24,29,37,38]. These findings are presented alongside comparative biomarker data in Table 1.

#### 4.3.3. Ketogenic Diet and Iron Metabolism in Obese Individuals

No published study has directly examined iron deficiency as a primary outcome in obese human participants undergoing a ketogenic dietary intervention. The evidence synthesized in this section derives from: (i) mechanistic studies of high-fat diet effects on intestinal iron transport proteins in animal and translational models; (ii) secondary analyses of ketogenic diet trials that reported iron biomarkers; and (iii) dietary composition analyses comparing ketogenic diets with standard recommendations for iron intake. This absence of direct human evidence is a key gap requiring targeted prospective investigation (see Section 5.4). The ketogenic diet restricts carbohydrate intake to <50 g/day, inducing nutritional ketosis through fat oxidation and hepatic ketone body production [15]. This necessitates elimination or severe restriction of many iron-rich plant foods including legumes, whole grains, most fruits, and starchy vegetables—foods that collectively represent major non-heme iron sources in typical Western diets [15].

A key mechanistic concern with ketogenic diets is the potential for hepcidin-independent impairment of iron absorption. Animal and translational studies have demonstrated that high dietary fat intake reduces duodenal expression of DMT1 and ferroportin, the primary iron transport proteins in the intestinal epithelium [15]. This effect appears to be independent of hepcidin and may represent a direct effect of lipid-mediated changes in enterocyte membrane composition and transporter trafficking [15].

Counterbalancing these risks, ketogenic diets typically include iron-rich animal foods such as red meat, poultry, and fish, which provide highly bioavailable heme iron. Moreover, the rapid weight loss induced by ketogenic diets may reduce adipose-driven inflammation and hepcidin. However, PUFA supplementation studies suggest that the type of dietary fat matters: *n*-3 PUFA supplementation during caloric restriction attenuated hepcidin elevation and preserved iron status compared to *n*-6 PUFA or saturated fat-enriched diets [37,38].

The net effect of ketogenic diets on iron status in obese individuals remains insufficiently characterized. The restriction of dietary iron diversity, combined with potential hepcidin-independent absorption impairment, suggests that ketogenic diets may pose the highest risk of iron deficiency among the three dietary patterns examined.

Quantitative iron biomarker data from ketogenic diet studies included in this review indicated serum ferritin reductions of 8–15% from baseline values of 85–128 μg/L. Hemoglobin concentrations showed modest declines of 0.2–0.6 g/dL from baseline values of 13.2–14.5 g/dL, consistent with a degree of functional iron restriction. IL-6 concentrations were reduced by 15–30% from baseline values of 3.1–7.2 pg/mL [15,23,24,25]. A comparative summary of these measurements across dietary patterns is provided in Table 1.

#### 4.3.4. Intermittent Fasting and Iron Status in Obese Individuals

Intermittent fasting encompasses several protocols including time-restricted feeding (e.g., 16:8 or 18:6), alternate-day fasting, and periodic prolonged fasting [16]. These approaches induce metabolic remodeling through cyclical transitions between fed and fasted states, activating autophagy, reducing insulin resistance, and promoting weight loss [16].

A comprehensive multi-omic study of intermittent fasting revealed complex remodeling of iron metabolism during fasting periods [16]. Fasting acutely elevated serum transferrin levels (consistent with increased iron transport capacity) while decreasing ferritin (suggesting mobilization of iron stores), with both changes remaining within physiological reference ranges [16]. The acute hypoferremic response to short-term fasting—characterized by elevated hepcidin and reduced serum iron—is a well-documented physiological adaptation that may be misinterpreted as pathological iron deficiency if assessed during fasting periods [16].

The long-term effects of intermittent fasting on iron status appear to be mediated primarily through weight loss and inflammation reduction. Studies of caloric restriction with intermittent fasting elements have demonstrated reductions in serum ferritin and hepcidin following significant weight loss [24]. A critical consideration is the quality of the diet during eating windows: if characterized by iron-poor dietary choices, intermittent fasting may exacerbate iron deficiency risk despite its beneficial effects on inflammation and weight.

Quantitative assessment of iron biomarkers from intermittent fasting studies in this review demonstrated serum ferritin reductions of 12–22% from baseline concentrations of 78–108 μg/L. Hemoglobin values remained within clinically stable ranges, with baselines of 13.0–14.1 g/dL and changes not exceeding ±0.3 g/dL. IL-6 levels declined by 22–38% from baseline concentrations of 2.9–5.8 pg/mL [16,24]. A comprehensive summary of these quantitative measurements is presented in Table 1.

#### 4.3.5. Comparative Analysis: Which Diet Poses the Greatest Iron Deficiency Risk

Based on the synthesis of available evidence, a hierarchy of iron deficiency risk can be proposed for the three dietary patterns in obese individuals [3,15,16]. The Mediterranean diet appears most protective for iron status due to its strong anti-inflammatory profile [14,37,38], abundant non-heme iron sources [3,14,29], high vitamin C content [17], and demonstrated improvements in iron biomarkers in clinical trials [24,29]. The ketogenic diet may pose the highest risk due to restricted dietary iron diversity from plant sources [15] and potential hepcidin-independent impairment of intestinal iron transport [15]; however, this conclusion is based primarily on mechanistic and animal data and requires confirmation in human trials. Intermittent fasting occupies an intermediate position [16], with complex acute and chronic effects [2,16] strongly modulated by the quality of the diet during eating windows [3,24].

Critically, weight loss per se consistently improves iron homeostasis in obese individuals by reducing adipose-driven inflammation and lowering hepcidin [24,25]. The differential risk profile is therefore most relevant in the early phases of dietary intervention and in individuals with pre-existing iron deficiency or at high risk (e.g., premenopausal women, individuals with inflammatory bowel disease, or those with prior bariatric surgery). Table 2 summarizes the comparative effects of the three dietary patterns on key iron metabolism parameters in obese individuals.

A comprehensive comparison of quantitative biomarker data across all three dietary patterns, including serum ferritin, hemoglobin, IL-6, and hepcidin measurements stratified by dietary intervention, is provided in Table 1.

## 5. Discussion

### 5.1. Summary of Findings

This systematic literature review provides the first comprehensive synthesis comparing the effects of the Mediterranean diet, ketogenic diet, and intermittent fasting on iron deficiency risk in obese individuals. The central finding is that these three dietary patterns differ substantially in their effects on iron metabolism, with the Mediterranean diet appearing most protective and the ketogenic diet potentially posing the highest risk.

The quantitative biomarker data assembled in Table 1 provide concrete physiological evidence corroborating the comparative risk hierarchy proposed above. Mediterranean diet interventions produced the most favorable iron biomarker profile, with the largest IL-6 reductions (28–42%) and fully preserved hemoglobin levels, consistent with the anti-inflammatory mechanisms underpinning this dietary pattern’s protective effect on iron status. By contrast, ketogenic diet studies demonstrated measurable hemoglobin declines (0.2–0.6 g/dL) alongside the smallest IL-6 reductions (15–30%), reflecting the competing influences of reduced adipose-driven inflammation, hepcidin suppression, and impaired duodenal iron absorption characteristic of very-low-carbohydrate diets. Intermittent fasting exhibited intermediate outcomes across all biomarkers—ferritin reductions of 12–22%, stable hemoglobin, and IL-6 decreases of 22–38%—placing it between the Mediterranean and ketogenic patterns in terms of iron metabolic risk. Hepcidin measurements, available in a subset of studies, aligned directionally with the ferritin and IL-6 trajectories across all three patterns, reinforcing the centrality of the hepcidin–ferroportin axis in mediating the differential iron metabolic effects of these dietary interventions.

The hepcidin-centric model of obesity-related iron deficiency provides the mechanistic framework for understanding these differential effects. In obesity, chronic inflammation drives hepcidin elevation, which blocks iron absorption and sequestration, leading to functional iron deficiency [9,10,11]. Dietary patterns that reduce inflammation—such as the Mediterranean diet—may attenuate hepcidin stimulation and preserve iron homeostasis. Conversely, dietary patterns that restrict iron-rich foods or impair intestinal iron transport—as potentially occurs with ketogenic diets—may exacerbate iron deficiency risk in this already vulnerable population.

The finding that weight loss per se consistently improves iron status across all dietary patterns highlights the primacy of adiposity reduction in restoring iron homeostasis [24,25]. This suggests that all three dietary patterns may ultimately improve iron status if sufficient weight loss is achieved and maintained. The differential risk profile is therefore most relevant in the early phases of dietary intervention, before significant weight loss has occurred, and in individuals with pre-existing iron deficiency or at high risk.

The role of specific dietary components in modulating iron absorption merits emphasis. Vitamin C from fruits and vegetables enhances non-heme iron absorption by reducing ferric (Fe3+) to ferrous (Fe2+) iron and forming soluble iron chelates [17]. The Mediterranean diet’s abundance of vitamin C-rich foods may therefore provide a practical advantage in maintaining iron absorption despite elevated hepcidin in obese individuals. The Mankai duckweed-enriched green-Mediterranean diet demonstrated particularly impressive iron status improvements, suggesting that novel plant-based iron sources with high bioavailability may represent a promising dietary strategy [29].

For intermittent fasting, the timing of iron assessment relative to fasting periods is critical. The acute hypoferremic response to fasting is a physiological adaptation that should not be misinterpreted as pathological iron deficiency [16]. Clinicians assessing iron status in patients following intermittent fasting protocols should ideally measure iron biomarkers during the eating phase to obtain accurate assessments of true iron stores.

### 5.2. Clinical Implications

Based on the evidence synthesized in this review, several clinical recommendations are proposed for managing iron status in obese individuals undergoing dietary interventions:

- Baseline iron status assessment: Before initiating any dietary intervention, iron biomarkers including serum iron, ferritin, transferrin saturation, and ideally hepcidin should be measured to identify pre-existing iron deficiency.
- Dietary iron monitoring: Patients following ketogenic diets should receive specific dietary counseling to ensure adequate intake of heme iron from animal sources.
- Vitamin C co-consumption: Patients following any dietary pattern should be encouraged to consume vitamin C-rich foods alongside iron-rich foods to enhance non-heme iron absorption.
- Periodic iron biomarker monitoring: Iron status should be reassessed at 3-month intervals during the initial weight loss phase, with more frequent monitoring in high-risk individuals (premenopausal women, individuals with prior bariatric surgery, or those with pre-existing iron deficiency).
- Fasting-phase timing: For patients following intermittent fasting, iron biomarker assessment should be scheduled during the eating phase to avoid misinterpretation of physiological hypoferremia.

### 5.3. Limitations

The absence of direct head-to-head RCTs comparing the Mediterranean diet, ketogenic diet, and intermittent fasting with iron status as a primary endpoint in obese individuals represents a fundamental gap in the evidence base. The comparative risk hierarchy proposed in this review is based on indirect evidence and mechanistic reasoning, and should be interpreted as hypothesis-generating only. The five-point eligibility scoring system used during screening was designed to operationalize the seven predefined criteria in a reproducible manner but is not a validated instrument; future reviews should adopt standard PICO-based screening with prospective registration (e.g., PROSPERO). The current review was not prospectively registered, which represents an additional limitation. Studies without accessible full-text PDFs were excluded after database and open-access searches, introducing potential selection bias. The inclusion of both primary studies and secondary sources (systematic reviews and meta-analyses) risks double-counting of primary study evidence; primary and secondary sources are distinguished in Supplementary Table S2 to allow readers to assess this potential source of evidence inflation. Evidence specific to Asian populations applying WHO/Asia–Pacific BMI thresholds (obesity class I: BMI 25.0–29.9 kg/m^2^) was not identified, limiting extrapolation to these populations.

The heterogeneity of included studies in terms of study design, population characteristics, dietary intervention protocols, iron biomarkers measured, and follow-up duration precluded formal meta-analysis. The predominance of observational studies limits causal inference. Potential confounders including sex, age, menopausal status, inflammatory status, baseline iron stores, concurrent medications, and dietary supplement use were incompletely reported across studies, limiting the ability to draw firm conclusions for specific subpopulations.

### 5.4. Future Research Directions

Well-designed RCTs directly comparing the Mediterranean diet, ketogenic diet, and intermittent fasting with iron status as a primary or secondary endpoint in obese adults are urgently needed. Such trials should measure a comprehensive panel of iron biomarkers including hepcidin, serum iron, ferritin, transferrin saturation, and transferrin receptor and should include stratified analyses by sex, menopausal status, and baseline iron status.

Mechanistic studies examining the effects of the ketogenic diet on duodenal iron transporter expression (DMT1, ferroportin) in human subjects are needed to confirm the hepcidin-independent absorption impairment suggested by animal studies. Investigation of novel iron-rich plant foods such as Mankai duckweed within different dietary frameworks could identify strategies to mitigate iron deficiency risk across dietary patterns.

## 6. Conclusions

This systematic literature review synthesizes available—largely indirect and mechanistically derived—evidence on the differential effects of the Mediterranean diet, ketogenic diet, and intermittent fasting on iron metabolism and iron deficiency risk in adults with obesity. In the absence of direct head-to-head comparative trials, all conclusions should be regarded as hypothesis-generating and provisional. On the basis of available mechanistic, observational, and indirect clinical evidence, the Mediterranean diet appears most protective for iron status, attributable to its anti-inflammatory profile (which may attenuate hepcidin stimulation), abundant non-heme iron sources co-consumed with vitamin C-rich foods, and demonstrated improvements in iron biomarkers in clinical trials [24,29]. The ketogenic diet may carry the highest risk of iron deficiency due to restricted dietary iron diversity from plant sources and potential hepcidin-independent impairment of intestinal iron absorption [15]; however, this conclusion rests primarily on mechanistic and animal data, and direct human evidence is lacking. Intermittent fasting occupies an intermediate and complex position, with outcomes dependent on the nutritional quality of the diet during eating windows [2,16].

A unifying finding across all dietary patterns is that weight loss per se consistently improves iron homeostasis in obese individuals by reducing adipose-driven inflammation and lowering hepcidin. This underscores the central role of adiposity reduction in restoring iron metabolism.

Given the absence of direct comparative RCTs, the risk hierarchy proposed in this review should be interpreted as hypothesis-generating rather than definitive. Detailed data extraction for all 38 included studies—including sample sizes, study duration, population characteristics, iron biomarkers measured, and main findings—are available in Supplementary Table S2. The PRISMA 2020 checklist is provided in Supplementary Table S1, and complete database search strings are provided in Supplementary Table S3. Clinicians should routinely monitor iron biomarkers—including hepcidin where available—in obese patients undertaking any dietary intervention, with particular vigilance in high-risk populations including premenopausal women and individuals with pre-existing iron deficiency. Future research should prioritize head-to-head dietary trials with iron status as a primary endpoint to definitively establish the comparative risk profiles of these increasingly popular dietary approaches.

## Figures and Tables

**Table 1:** Quantitative biomarker data stratified by dietary pattern in obese adults (synthesis from included studies).

Biomarker	Mediterranean Diet	Ketogenic Diet	Intermittent Fasting
Studies reporting (*n*)	12	8	7
Serum ferritin μg/L baseline	72–115	85–128	78–108
Ferritin post-intervention change	−18–28%	−8–15%	−12–22%
Hemoglobin g/dL baseline	12.9–14.2	13.2–14.5	13.0–14.1
Hemoglobin change g/dL	≤±0.2	−0.2 to −0.6	≤±0.3
IL-6 pg/mL baseline	2.8–6.4	3.1–7.2	2.9–5.8
IL-6 change	−28–42%	−15–30%	−22–38%
Hepcidin ng/mL baseline	45–82	52–95	48–88
Hepcidin change	−20–35%	−10–20%	−15–28%

**Table 2:** Comparative effects of the Mediterranean diet, ketogenic diet, and intermittent fasting on iron metabolism parameters in obese individuals.

Parameter	Mediterranean Diet	Ketogenic Diet	Intermittent Fasting	Supporting References
Dietary iron content	High (legumes, leafy greens, fish, nuts)	Moderate (red meat, poultry); restricted plant sources	Depends on eating window diet quality	[3,14,15,16,17]
Iron bioavailability	Enhanced by vitamin C, polyphenols; Mankai duckweed	High heme iron; potentially impaired non-heme absorption	Variable; acute fasting may reduce absorption	[15,17,29]
Anti-inflammatory effect	Strong (omega-3, polyphenols, fiber)	Moderate (via ketosis and weight loss)	Moderate (via weight loss and metabolic remodeling)	[14,37,38]
Hepcidin effect	Decreased (anti-inflammatory)	Decreased via weight loss; potentially increased via high fat	Acute increase during fasting; chronic decrease via weight loss	[9,10,15,16,24]
Serum iron	Increased or preserved	Uncertain; potentially decreased	Acute decrease; chronic improvement with weight loss	[24,29]
Ferritin	Decreased (inflammation reduction)	Decreased via weight loss	Decreased (iron mobilization and weight loss)	[24,25,26]
Overall iron deficiency risk	LOW (most protective)	HIGH (most risk)	INTERMEDIATE	[3,15,16,24]

## Data Availability

No new data were created or analyzed in this study. Data sharing is not applicable to this article.

## Supplementary Materials

The following supporting information can be downloaded at https://www.mdpi.com/article/10.3390/nu18162681/s1. Table S1: PRISMA 2020 checklist for systematic reviews. Table S2: Full Data Extraction Table for All 38 Included Studies. Table S3: Complete search strings and filters used for all databases.

## Abbreviations

BMI	Body Mass Index
DMT1	Divalent Metal Transporter 1
IL-6	Interleukin-6
JAK2	Janus Kinase 2
PICO	Population, Intervention, Comparison, Outcome
PRISMA	Preferred Reporting Items for Systematic Reviews and Meta-Analyses
PUFA	Polyunsaturated Fatty Acid
RCT	Randomized Controlled Trial
STAT3	Signal Transducer and Activator of Transcription 3
TIBC	Total Iron-Binding Capacity
TNF-alpha	Tumor Necrosis Factor-alpha
