# Supplementary material for: Effects of the Mediterranean Diet, Ketogenic Diet, and Intermittent Fasting on Iron Metabolism and Iron Deficiency Risk in Adults with Obesity: A Systematic Literature Review
Source: Nutrients. 2026 Aug 17;18(16):2681. doi: 10.3390/nu18162681 (PMC13517051; doi:10.3390/nu18162681)
Supplement: Supplementary file 1 [file nutrients-18-02681-s001.zip › Supplementary_Table_S1_PRISMA_2020_Checklist.pdf]

Supplementary Materials

Table S1. PRISMA 2020 Checklist for Systematic Reviews

Section and Topic	Item #	Checklist Item	Location in Manuscript
**TITLE**			
Title	1	Identify the report as a systematic review.	Title, p. 1
**ABSTRACT**			
Abstract	2	See the PRISMA 2020 for Abstracts checklist.	Abstract, p. 1
**INTRODUCTION**			
Rationale	3	Describe the rationale for the review in the context of existing knowledge.	Section 1 (Introduction), pp. 1–2
Objectives	4	Provide an explicit statement of the objective(s) or question(s) the review addresses.	Section 1 (Introduction), pp. 1–2
**METHODS**			
Eligibility criteria	5	Specify the inclusion and exclusion criteria for the review and how studies were grouped for the syntheses.	Section 3 (Methods), pp. 4–5
Information sources	6	Specify all databases, registers, websites, organisations, reference lists and other sources searched or consulted to identify studies.	Section 3 (Methods), pp. 4–5
Search strategy	7	Present the full search strategies for all databases, registers and websites, including any filters and limits used.	Section 3 (Methods), pp. 4–5
Selection process	8	Specify the methods used to decide whether a study met the inclusion criteria of the review, including how many reviewers screened each record and each report retrieved, whether they worked independently, and if applicable, details of automation tools used in the process.	Section 3 (Methods), pp. 4–5
Data collection process	9	Specify the methods used to collect data from reports, including how many reviewers collected data from each report, whether they worked independently, any processes for obtaining or confirming data from study	Section 3 (Methods), pp. 4–5

Section and Topic	Item #	Checklist Item	Location in Manuscript
		investigators, and if applicable, details of automation tools used in the process.	
Data items	10a	List and define all outcomes for which data were sought. Specify whether all results that were compatible with each outcome domain in each study were sought (e.g. for all measures, time points, analyses), and if not, the methods used to decide which results to collect.	Section 3 (Methods), pp. 4–5
Data items	10b	List and define all other variables for which data were sought (e.g. participant and intervention characteristics, funding sources). Describe any assumptions made about any missing or unclear information.	Section 3 (Methods), pp. 4–5
Study risk of bias assessment	11	Specify the methods used to assess risk of bias in the included studies, including details of the tool(s) used, how many reviewers assessed each study and whether they worked independently, and if applicable, details of automation tools used in the process.	Not reported
Effect measures	12	Specify for each outcome the effect measure(s) (e.g. risk ratio, mean difference) used in the synthesis or presentation of results.	Not applicable
Synthesis methods	13a	Describe the processes used to decide which studies were eligible for each synthesis (e.g. tabulating the study intervention characteristics and comparing against the planned groups for each synthesis (item #5)).	Section 3 (Methods), pp. 4–5
Synthesis methods	13b	Describe any methods required to prepare the data for presentation or synthesis, such as handling of missing summary statistics, or data conversions.	Not applicable
Synthesis methods	13c	Describe any methods used to tabulate or visually display results of individual studies and syntheses.	Section 4 (Results), pp. 5–9; Table 1
Synthesis methods	13d	Describe any methods used to synthesise results and provide a rationale for the choice(s). If meta-analysis was performed, describe the model(s), method(s) to identify the presence and extent of statistical heterogeneity, and software package(s) used.	Not applicable (narrative synthesis)

Section and Topic	Item #	Checklist Item	Location in Manuscript
Synthesis methods	13e	Describe any methods used to explore possible causes of heterogeneity among study results (e.g. subgroup analysis, meta-regression).	Not applicable
Synthesis methods	13f	Describe any sensitivity analyses conducted to assess robustness of the synthesised results.	Not applicable
Reporting bias assessment	14	Specify any methods used to assess risk of bias due to missing results in a synthesis (arising from reporting biases).	Not reported
Certainty assessment	15	Specify any methods used to assess certainty (or confidence) in the body of evidence for an outcome.	Not applicable
**RESULTS**			
Study selection	16a	Describe the results of the search and selection process, from the number of records identified in the search to the number of studies included in the review, ideally using a flow diagram.	Section 4.1 (Results), pp. 5–6; Figure S1
Study selection	16b	Cite each included study.	Section 4.2–4.3, pp. 6–9; References
Study characteristics	17	Present the characteristics of each included study.	Section 4 (Results), pp. 5–9; Table 1
Risk of bias in studies	18	Present assessments of risk of bias for each included study.	Not assessed
Results of individual studies	19	For all outcomes, present, for each study: (a) summary statistics for each group (where appropriate) and (b) an effect estimate and its precision (e.g. confidence/credible interval), ideally using structured tables or plots.	Section 4 (Results), pp. 5–9; Table 1
Results of syntheses	20a	For each synthesis, briefly summarise the characteristics and risk of bias among contributing studies.	Not applicable
Results of syntheses	20b	Present results of all statistical syntheses conducted. If meta-analysis was done, present for each the summary estimate and its precision (e.g. confidence/credible interval) and measures of statistical heterogeneity. If comparing groups, describe the direction of the effect.	Not applicable

Section and Topic	Item #	Checklist Item	Location in Manuscript
Results of syntheses	20c	Present results of all investigations of possible causes of heterogeneity among study results.	Not applicable
Results of syntheses	20d	Present results of all sensitivity analyses conducted to assess the robustness of the synthesised results.	Not applicable
Reporting biases	21	Present assessments of risk of bias due to missing results (arising from reporting biases) for each synthesis assessed.	Not assessed
Certainty of evidence	22	Present assessments of the certainty (or confidence) in the body of evidence for each outcome assessed.	Not assessed
**DISCUSSION**			
Discussion	23a	Provide a general interpretation of the results in the context of other evidence.	Section 5 (Discussion), pp. 10–12
Discussion	23b	Discuss any limitations of the evidence included in the review.	Sections 5–6, pp. 12–13
Discussion	23c	Discuss any limitations of the review processes used.	Sections 5–6, pp. 12–13
Discussion	23d	Discuss implications of the results for practice, policy, and future research.	Sections 5–6, pp. 12–13
**OTHER INFORMATION**			
Registration and protocol	24a	Provide registration information for the review, including register name and registration number, or state that the review was not registered.	Not pre-registered
Registration and protocol	24b	Indicate where the review protocol can be accessed, or state that a protocol was not prepared.	Protocol not prepared
Registration and protocol	24c	Describe and explain any amendments to information provided at registration or in the protocol.	Not applicable
Support	25	Describe sources of financial or other support for the review, and the role of the funders or sponsors in the review.	Funding, p. 1

Section and Topic	Item #	Checklist Item	Location in Manuscript
Competing interests	26	Declare any competing interests of review authors.	Conflicts of Interest, p. 1
Availability of data, code, and other materials	27	Report which of the following are publicly available and where they can be found: template data collection forms; data extracted from included studies; data used for all analyses; analytic code; any other materials used in the review.	Not applicable
