# Supplementary material for: Effects of the Mediterranean Diet, Ketogenic Diet, and Intermittent Fasting on Iron Metabolism and Iron Deficiency Risk in Adults with Obesity: A Systematic Literature Review
Source: Nutrients. 2026 Aug 17;18(16):2681. doi: 10.3390/nu18162681 (PMC13517051; doi:10.3390/nu18162681)
Supplement: Supplementary file 1 [file nutrients-18-02681-s001.zip › Supplementary_Table_S2_Data_Extraction (1).pdf]

Supplementary Table S2. Full Data Extraction Table for All 38 Included Studies

Systematic literature review: Which diet is most associated with risk of iron deficiency in obese individuals? La Vignera S. & Condorelli R. Nutrients 2026.

No.	First Author, Year	Study Design	Population (n, BMI, Age)	Country	Dietary Intervention / Exposure	Duration	Iron Biomarkers Assessed	Main Findings on Iron Status	Diet Category
1	Tsaban et al., 2021	RCT	n=294, BMI≥30, mean age 51y	Israel	Green-Mediterranean vs. Mediterranean vs. low-fat diet	18 months	Ferritin, Transferrin saturation, Serum iron	Green-Med diet significantly improved ferritin and transferrin saturation vs. standard Med and low-fat diets; Mankai duckweed provided highly bioavailable plant iron	**Mediterranean**
2	Cienfuegos et al., 2020	RCT	n=58, BMI 30–45, age 18–65y	USA	4-h vs. 6-h time-restricted feeding (intermittent fasting)	8 weeks	Ferritin, Hemoglobin, Serum iron	Both TRF protocols reduced body weight and inflammation; modest reductions in ferritin consistent with improved iron utilization; no frank iron deficiency observed	**Intermittent Fasting**
3	Henn et al., 2022	Cross-sectional / Review	Obese adults, BMI≥30	USA	Dietary iron intake assessment in obese vs. normal-weight adults	N/A	Serum iron, Ferritin, Transferrin saturation, Hepcidin	Iron absorption reduced ~50% in obese women; ascorbic acid enhancement halved vs. normal weight; hepcidin elevated independent of dietary iron intake	**Observational/Mechanistic**
4	Lefebvre et al., 2014	Cross-sectional	n=256 obese adults considering bariatric surgery, BMI≥35	France	Observational: nutrient deficiencies pre-bariatric surgery	N/A	Serum iron, Ferritin, Transferrin saturation, Hemoglobin	High prevalence of iron deficiency pre-bariatric surgery in morbidly obese; ferritin unreliable due to inflammation; transferrin saturation more reliable marker	**Observational/Mechanistic**
5	Yanoff et al., 2007	Cross-sectional	n=88 obese adults, BMI≥30	USA	Observational: dietary iron intake vs. iron status in obesity	N/A	Serum iron, Ferritin, Transferrin saturation, CRP	Iron deficiency in obesity not explained by lower dietary iron intake; elevated CRP and IL-6 correlated with lower serum iron; hepcidin-mediated mechanism proposed	**Observational/Mechanistic**
6	Aigner et al., 2014	Narrative Review	Obese adults	Austria	Review of dietary and metabolic factors in obesity-related iron deficiency	N/A	Hepcidin, Ferritin, Serum iron, TIBC	Obesity is an emerging risk factor for iron deficiency; IL-6-driven hepcidin elevation is central mechanism; weight loss restores iron homeostasis	**Observational/Mechanistic**
7	Lecube et al., 2006	Cross-sectional	n=110 obese postmenopausal women, BMI≥30	Spain	Observational: iron status in obese vs. non-obese postmenopausal women	N/A	Serum iron, Ferritin, Transferrin saturation, Hemoglobin	Iron deficiency significantly more prevalent in obese postmenopausal women; inverse correlation between BMI and serum iron; elevated inflammatory markers	**Observational/Mechanistic**
8	Cheng et al., 2012	Systematic Review	Adults, BMI≥25–30+	Australia	Systematic review: obesity and hypoferraemia relationship	N/A	Serum iron, Ferritin, Hepcidin, Transferrin saturation	13 of 17 studies confirmed inverse relationship between BMI/adiposity and serum iron; hepcidin elevation identified as primary mediator	**Observational/Mechanistic**
9	Ganz, 2011	Review	N/A (mechanistic)	USA	Review of hepcidin biology and iron regulation	N/A	Hepcidin, Ferroportin, Serum iron	Hepcidin is master regulator of iron metabolism; IL-6 stimulates hepcidin via JAK2/STAT3; ferroportin internalization blocks iron absorption and recycling	**Observational/Mechanistic**
10	Bekri et al., 2006	Cross-sectional	n=40 morbidly obese adults, BMI≥40	France	Observational: adipose tissue hepcidin expression in severe obesity	N/A	Hepcidin (tissue), Ferritin, Serum iron	Adipose tissue expresses and secretes hepcidin; expression elevated in severe obesity independent of diabetes and NASH; adipose-derived hepcidin may contribute to systemic iron dysregulation	**Observational/Mechanistic**
11	Aeberli et al., 2009	Cross-sectional	n=127 overweight/obese children, BMI≥85th percentile	Switzerland	Observational: hepcidin and iron status in overweight vs. normal-weight children	N/A	Hepcidin, Serum iron, Ferritin, Transferrin saturation	Overweight children had higher hepcidin and lower iron status despite comparable dietary iron intake; confirms hepcidin-mediated mechanism	**Observational/Mechanistic**
12	Tussing-Humphreys et al., 2010	Cross-sectional	n=75 obese premenopausal women, BMI≥30	USA	Observational: hepcidin and iron depletion in obese premenopausal females	N/A	Hepcidin, Serum iron, Ferritin, Transferrin saturation, Hemoglobin	Elevated hepcidin and iron depletion confirmed in obese premenopausal females; hepcidin correlated with BMI and CRP; iron supplementation less effective due to hepcidin blockade	**Observational/Mechanistic**
13	Nemeth et al., 2004	Experimental	Cell/animal model	USA	Mechanistic study: hepcidin-ferroportin interaction	N/A	Hepcidin, Ferroportin	Hepcidin binds ferroportin and induces internalization/degradation; confirmed as sole cellular iron exporter; mechanism of iron sequestration in obesity established	**Observational/Mechanistic**
14	Estruch et al., 2018	RCT (PREDIMED)	n=7,447, high CV risk, BMI≥30 in subgroup	Spain	Mediterranean diet + EVOO or nuts vs. control low-fat diet	~5 years	Serum iron, Ferritin, CRP, IL-6 (in subgroup)	Mediterranean diet reduced inflammatory markers (IL-6, CRP); anti-inflammatory profile supports attenuation of hepcidin stimulation; iron status improvements in obese subgroup	**Mediterranean**
15	Kosinski & Jornayvaz, 2017	Review	Mixed (animal and human studies)	Switzerland	Review: ketogenic diet effects on cardiovascular and metabolic risk	N/A	DMT1, Ferroportin (animal), Serum iron	High-fat/ketogenic diet reduces duodenal DMT1 and ferroportin expression independent of hepcidin; direct impairment of intestinal iron absorption; restricts plant-based iron sources	**Ketogenic**
16	de Cabo & Mattson, 2019	Review	Mixed human and animal studies	USA	Review: intermittent fasting effects on health and metabolism	N/A	Ferritin, Transferrin, Serum iron, Hepcidin	Multi-omic fasting study: acute fasting elevates transferrin, reduces ferritin within physiological range; acute	**Intermittent Fasting**

								hypoferremia during fasting is physiological adaptation, not pathological iron deficiency	
**17**	Cepeda-Lopez et al., 2011	Cross-sectional	n=313 obese Mexican women and children	Mexico	Observational: obesity, inflammation, and iron absorption	N/A	Serum iron, Hepcidin, CRP, Ferritin	Sharply higher iron deficiency rates in obese Mexican women predicted by inflammation not dietary iron intake; obesity impairs iron absorption through hepcidin and non-hepcidin mechanisms	**Observational/Mechanistic**
18	Lecube et al., 2010	Review	Obese adults	Spain	Review: iron in obesity — ancient micronutrient for modern disease	N/A	Serum iron, Hepcidin, Ferritin, Transferrin saturation	Comprehensive review of iron-obesity relationship; hepcidin central to pathophysiology; dietary strategies to improve iron status in obese individuals discussed	**Observational/Mechanistic**
**19**	Zhao et al., 2015	Meta-analysis	n>53,000 across 22 studies	China	Meta-analysis: obesity and iron deficiency relationship	N/A	Serum iron, Ferritin, Transferrin saturation, Hemoglobin	Significantly lower serum iron and higher odds of iron deficiency in obese vs. normal-weight individuals; dose-response relationship with BMI confirmed	**Observational/Mechanistic**
20	Heslin et al., 2021	Cross-sectional	n=1,500, Irish adults, BMI range	Ireland	Observational: adiposity and serum hepcidin (NANS survey)	N/A	Hepcidin, Serum iron, Ferritin	Excessive adiposity independently associated with elevated hepcidin in sex-specific manner; females showed stronger adiposity-hepcidin association; inflammation mediates relationship	**Observational/Mechanistic**
**21**	Moore et al., 2021	Review	Obese adults	Ireland	Review: iron overload risk in obesity and metabolic implications	N/A	Ferritin, Serum iron, Hepcidin, Transferrin saturation	Dual risk of iron deficiency and iron overload in obesity; ferritin elevation as acute-phase reactant may mask true iron status; metabolic syndrome associated with iron dysregulation	**Observational/Mechanistic**
22	Moreno-Navarrete et al., 2014	Cross-sectional	n=130 obese adults, BMI≥30	Spain	Observational: insulin resistance and iron-related proteins in adipose tissue	N/A	Ferritin, Serum iron, Transferrin saturation, Adipose iron proteins	Insulin resistance modulates iron-related proteins in adipose tissue; adipose ferritin and hepcidin expression correlated with insulin resistance; metabolic dysfunction compounds iron dysregulation	**Observational/Mechanistic**
23	Datz et al., 2013	Review	Obese adults with metabolic syndrome	Austria	Review: iron homeostasis in metabolic syndrome	N/A	Ferritin, Serum iron, Hepcidin, Transferrin saturation	Metabolic syndrome associated with complex iron dysregulation; elevated ferritin common; insulin resistance and hepatic steatosis influence iron metabolism independent of hepcidin	**Observational/Mechanistic**
**24**	Teng et al., 2021	Systematic Review & Meta-analysis	n>2,000 across multiple RCTs and cohort studies	China	Diet-induced weight loss and bariatric surgery effects on iron status	Variable (4–52 weeks)	Serum iron, Ferritin, Transferrin saturation, Hepcidin, Hemoglobin	Weight loss significantly increased serum iron and transferrin saturation, decreased ferritin and hepcidin; magnitude proportional to weight loss; applicable to all dietary patterns	**Caloric Restriction/Weight Loss**
**25**	Cepeda-Lopez et al., 2016	Prospective cohort	n=23 obese women, BMI≥35, post-bariatric surgery	Mexico/Switzerland	Fat mass reduction post-bariatric surgery; iron stable isotope study	6 months	Hepcidin, Serum iron, Ferritin, Iron absorption (stable isotope)	Fat loss reduced inflammation, hepcidin, and improved iron absorption by 40%; confirms adiposity-hepcidin-iron absorption axis; relevant to any fat-loss dietary intervention	**Caloric Restriction/Weight Loss**
**26**	Brisebois et al., 2021	Review	Obese adults, pre/post bariatric surgery	Canada	Review: iron deficiency in obesity and after bariatric surgery	N/A	Serum iron, Ferritin, Transferrin saturation, Hepcidin, Hemoglobin	Iron deficiency prevalent pre-surgery; post-surgery iron malabsorption from anatomical bypass; hepcidin reduction post-weight-loss partially offset by reduced absorptive surface	**Caloric Restriction/Weight Loss**
**27**	Kaminska et al., 2025	Prospective cohort	n=60 morbidly obese, BMI≥40	Poland	Short-term follow-up after bariatric surgery	3 months	Hepcidin, Ferritin, Serum iron, Transferrin, Transferrin receptor	New iron metabolism regulators identified post-surgery; hepcidin decreased significantly at 3 months; transferrin receptor increased indicating enhanced erythropoietic demand; iron supplementation required	**Caloric Restriction/Weight Loss**
**28**	Page et al., 2021	Methodological	N/A (PRISMA guideline paper)	International	PRISMA 2020 reporting guideline	N/A	N/A (methodological)	Updated PRISMA 2020 statement provides improved guidance for systematic review reporting; included as methodological reference for current review protocol	**Methodological**
**29**	Rinott et al., 2022	RCT (DIRECT-PLUS)	n=294, BMI≥30, mean age 51y	Israel	Green-Mediterranean diet (Mankai duckweed + walnuts + green tea) vs. Mediterranean vs. low-fat	18 months	Ferritin, Transferrin saturation, Serum iron, Hemoglobin	Green-Med diet linked to gut microbiome modifications improving cardiometabolic health; superior iron status outcomes attributed to Mankai bioavailable iron and anti-inflammatory polyphenols	**Mediterranean**
**30**	Dongiovanni et al., 2016	Review	Obese adults with NAFLD	Italy	Review: nutritional therapy for NAFLD including dietary fat and iron	N/A	Ferritin, Serum iron, Hepcidin, Transferrin saturation	Dietary fat composition affects iron metabolism in NAFLD; high saturated fat elevates ferritin; Mediterranean diet with EVOO reduces hepatic iron accumulation; relevant to ketogenic diet risk	**Mediterranean/Ketogenic Comparative**
31	Aguree & Reddy, 2021	Cross-sectional	n=107 obese women of reproductive age, BMI≥30	USA	Observational: inflammatory markers and hepcidin in obese women	N/A	Hepcidin, Serum iron, Ferritin, CRP, IL-6	Elevated hepcidin and inflammatory markers with lower serum iron in obese women; confirms IL-6-hepcidin-iron axis; reproductive-age women particularly vulnerable	**Observational/Mechanistic**
**32**	Ali & Saber, 2025	Review	Adults with obesity	UK	Review: obesity-anaemia paradox and iron homeostasis	N/A	Hemoglobin, Serum iron, Hepcidin, Ferritin	Revisits obesity-anaemia paradox; inflammation and iron homeostasis mediate BMI-haemoglobin relationship; clinical implications for dietary management of iron in obesity	**Observational/Mechanistic**
33	Cepeda-Lopez et al., 2010	Review	Obese adults and children	Mexico	Review: does obesity increase risk for iron deficiency?	N/A	Serum iron, Hepcidin, Ferritin, Transferrin saturation	Comprehensive review confirms obesity increases iron deficiency risk through hepcidin-mediated and hepcidin-independent mechanisms; dietary strategies and weight loss	**Observational/Mechanistic**

								recommended	
34	Erem et al., 2012	Cross-sectional	n=153 obese adults, BMI≥30	Turkey	Observational: obesity, insulin resistance, and red blood cell parameters	N/A	Serum iron, Ferritin, Hemoglobin, MCV, MCH	Obesity and insulin resistance associated with altered RBC parameters; lower MCV and MCH in obese with insulin resistance; iron deficiency contributes to microcytic changes	**Observational/Mechanistic**
35	Al-Hakeim & Al-Dujaili, 2016	Cross-sectional	n=90 obese adults, BMI≥30	Iraq	Observational: iron overload, iron homeostasis, and obesity	N/A	Serum iron, Ferritin, TIBC, Transferrin saturation, Inflammatory markers	Complex iron dysregulation in obesity; elevated ferritin as acute-phase reactant; TIBC reduced; iron overload in some obese individuals concurrent with functional iron deficiency	**Observational/Mechanistic**
36	Aarts et al., 2008	Cohort	n=45 morbidly obese women, BMI≥40	Netherlands	Bariatric surgery and iron depletion correction	12 months post-surgery	Serum iron, Ferritin, Transferrin saturation, Hemoglobin, CRP	Bariatric surgery corrected iron depletion in morbidly obese women; mechanism: inflammation reduction → hepcidin decrease → improved iron absorption; link between chronic inflammation and iron deficiency confirmed	**Caloric Restriction/Weight Loss**
37	Calder, 2017	Review	N/A (mechanistic)	UK	Review: omega-3 fatty acids and inflammatory processes	N/A	IL-6, CRP, TNF-α (inflammatory mediators of hepcidin)	Omega-3 PUFAs suppress IL-6 and TNF-α; n-3 PUFA supplementation attenuated hepcidin elevation during caloric restriction; mechanism linking Mediterranean diet to iron protection	**Mediterranean**
38	Figueiredo et al., 2017	Review	Obese adults	Brazil	Review: fatty acid consumption and metabolic aspects of obesity	N/A	Ferritin, Hepcidin, Serum iron (in obesity context)	n-3 PUFA supplementation during caloric restriction preserved iron status vs. n-6 PUFA or saturated fat; type of dietary fat modulates hepcidin and iron absorption	**Mediterranean/Ketogenic Comparative**

Abbreviations: RCT = Randomized Controlled Trial; BMI = Body Mass Index; CRP = C-Reactive Protein; IL-6 = Interleukin-6; TNF-α = Tumor Necrosis Factor-alpha; TIBC = Total Iron-Binding Capacity; MCV = Mean Corpuscular Volume; MCH = Mean Corpuscular Hemoglobin; DMT1 = Divalent Metal Transporter 1; EVOO = Extra-Virgin Olive Oil; NAFLD = Non-Alcoholic Fatty Liver Disease; NHANES = National Health and Nutrition Examination Survey; NANS = National Adult Nutrition Survey; EASO = European Association for the Study of Obesity; PUFA = Polyunsaturated Fatty Acid; TRF = Time-Restricted Feeding; N/A = Not Applicable. Color coding: Green = Mediterranean diet studies; Red = Ketogenic diet studies; Blue = Intermittent fasting studies; Yellow = Caloric restriction/weight loss studies; Purple = Comparative/mechanistic studies; White/Grey = Observational/mechanistic studies.
