# Supplementary material for: Effects of the Mediterranean Diet, Ketogenic Diet, and Intermittent Fasting on Iron Metabolism and Iron Deficiency Risk in Adults with Obesity: A Systematic Literature Review
Source: Nutrients. 2026 Aug 17;18(16):2681. doi: 10.3390/nu18162681 (PMC13517051; doi:10.3390/nu18162681)
Supplement: Supplementary file 1 [file nutrients-18-02681-s001.zip › Supplementary_Table_S3_Search_Strings (1).pdf]

Supplementary Table S3. Complete Search Strings and Filters Used for All Databases

Systematic literature review: Which diet is most associated with risk of iron deficiency in obese individuals? La Vignera S. & Condorelli R. Nutrients 2026.

A comprehensive literature search was performed across three databases (SciSpace, Google Scholar, and PubMed) through December 2025 with no restrictions on publication year. The search strategy employed controlled vocabulary (MeSH terms in PubMed) and free-text terms related to: (1) obesity, (2) iron deficiency and iron metabolism markers, and (3) three dietary patterns of interest: Mediterranean diet, ketogenic diet, and intermittent fasting. All searches were conducted by the two authors independently and cross-verified. Search results were exported and imported into the screening workflow for deduplication and eligibility assessment.

Database	Search String / Query	Filters Applied	Records Retrieved	Notes
**PubMed (U.S. National Library of Medicine)**				
**PubMed**	Search String 1 — Primary (MeSH + free-text):<br><br>(obesity[MeSH Terms] OR obese[Title/Abstract] OR "body mass index"[Title/Abstract] OR BMI[Title/Abstract] OR "overweight"[Title/Abstract]) AND ("iron deficiency"[MeSH Terms] OR "iron deficiency anemia"[MeSH Terms] OR ferritin[Title/Abstract] OR hepcidin[Title/Abstract] OR "serum iron"[Title/Abstract] OR "transferrin saturation"[Title/Abstract] OR "iron status"[Title/Abstract] OR "iron metabolism"[Title/Abstract] OR "iron homeostasis"[Title/Abstract] OR "iron absorption"[Title/Abstract]) AND ("Mediterranean diet"[Title/Abstract] OR "Mediterranean dietary pattern"[Title/Abstract] OR "ketogenic diet"[Title/Abstract] OR "low-carbohydrate diet"[Title/Abstract] OR "VLCKD"[Title/Abstract] OR "intermittent fasting"[Title/Abstract] OR "time-restricted feeding"[Title/Abstract] OR "time-restricted eating"[Title/Abstract] OR "alternate day fasting"[Title/Abstract])	Article types: All<br>Language: English<br>Date range: No restriction<br>Species: Human<br>Age: Adult (19+ years)	**n = 8**	Primary search; MeSH terms used for controlled vocabulary; human filter applied
**PubMed**	Search String 2 — Mechanistic/Hepcidin (MeSH + free-text):<br><br>(obesity[MeSH Terms] OR obese[Title/Abstract] OR adiposity[Title/Abstract]) AND (hepcidin[Title/Abstract] OR hepcidin[MeSH Terms] OR "iron regulatory hormone"[Title/Abstract]) AND ("dietary intervention"[Title/Abstract] OR "weight loss"[Title/Abstract] OR "caloric restriction"[Title/Abstract] OR "dietary pattern"[Title/Abstract])	Article types: All<br>Language: English<br>Date range: No restriction<br>Species: Human	**n = 4**	Supplementary mechanistic search; focused on hepcidin-diet interaction
**PubMed**	Search String 3 — Weight Loss & Iron (MeSH + free-text):<br><br>(obesity[MeSH Terms] OR obese[Title/Abstract]) AND ("weight loss"[MeSH Terms] OR "bariatric surgery"[MeSH Terms] OR "caloric restriction"[Title/Abstract]) AND ("iron deficiency"[MeSH Terms] OR ferritin[Title/Abstract] OR hepcidin[Title/Abstract] OR "serum iron"[Title/Abstract] OR "iron absorption"[Title/Abstract])	Article types: All<br>Language: English<br>Date range: 2004–2025<br>Species: Human	**n = 2**	Targeted search for weight loss-iron relationship; bariatric surgery studies included as mechanistic evidence

**PubMed TOTAL**	Three search strings combined; duplicates removed before pooling with other databases	Combined filters as above	**n = 14**	After combining 3 strings and removing within-database duplicates
**Google Scholar**				
**Google Scholar**	Search String 1 — Primary:<br><br>"obesity" AND "iron deficiency" AND ("Mediterranean diet" OR "ketogenic diet" OR "intermittent fasting")	Date range: All years<br>Language: English<br>Sort: Relevance<br>Cite count: No filter<br>Include patents: No<br>Include citations: No	**n = 22**	Top 22 most relevant results screened; Google Scholar does not support MeSH; free-text Boolean used
**Google Scholar**	Search String 2 — Hepcidin & Diet:<br><br>"hepcidin" AND "obesity" AND ("diet" OR "dietary intervention" OR "weight loss")	Date range: 2006–2025<br>Language: English<br>Sort: Relevance	**n = 15**	Supplementary hepcidin-focused search; top 15 relevant results
**Google Scholar**	Search String 3 — Iron Biomarkers & Obesity:<br><br>"iron status" AND "obese" AND ("ferritin" OR "transferrin saturation" OR "serum iron")	Date range: 2004–2025<br>Language: English<br>Sort: Relevance	**n = 12**	Targeted search for iron biomarker studies in obese populations
**Google Scholar**	Search String 4 — Mediterranean Diet & Iron:<br><br>"Mediterranean diet" AND ("iron" OR "ferritin" OR "anemia") AND "obesity"	Date range: All years<br>Language: English<br>Sort: Relevance	**n = 8**	Targeted Mediterranean diet-iron search; supplementary to primary
**Google Scholar TOTAL**	Four search strings combined; duplicates removed	Combined filters as above	**n = 57**	After combining 4 strings and removing within-database duplicates
**SciSpace (Typeset.io)**				
**SciSpace**	Search String 1 — Primary (AI-assisted semantic search):<br><br>"obesity iron deficiency Mediterranean diet ketogenic diet intermittent fasting"<br><br>Boolean equivalent:<br>(obesity OR obese) AND (iron deficiency OR ferritin OR hepcidin OR "iron status") AND ("Mediterranean diet" OR "ketogenic diet" OR "intermittent fasting")	Date range: All years<br>Language: English<br>Access: Open access preferred<br>Relevance threshold: ≥4.0/5.0 (abstract)<br>Full-text threshold: ≥4.5/5.0	**n = 180**	SciSpace AI-powered semantic search; relevance scoring applied; top results exported for screening
**SciSpace**	Search String 2 — Hepcidin & Dietary Patterns:<br><br>"hepcidin obesity dietary pattern iron metabolism"<br><br>Boolean equivalent:<br>(hepcidin) AND (obesity OR obese OR adiposity) AND ("dietary pattern" OR "dietary intervention" OR diet)	Date range: 2006–2025<br>Language: English<br>Relevance threshold: ≥4.0/5.0	**n = 75**	Supplementary hepcidin-focused search; SciSpace semantic ranking used
**SciSpace**	Search String 3 — Weight Loss & Iron Homeostasis:<br><br>"weight loss iron homeostasis obese adults"<br><br>Boolean equivalent:<br>("weight loss" OR "caloric restriction" OR "bariatric surgery") AND ("iron homeostasis" OR "iron status" OR ferritin OR hepcidin) AND (obesity OR obese)	Date range: 2008–2025<br>Language: English<br>Relevance threshold: ≥4.0/5.0	**n = 45**	Targeted weight loss-iron relationship search; bariatric and dietary studies included
**SciSpace**	Search String 4 — Ketogenic Diet & Iron:	Date range: All years	**n = 25**	Mechanistic ketogenic diet-

	"ketogenic diet iron absorption DMT1 ferroportin obesity"<br><br>Boolean equivalent:<br>("ketogenic diet" OR "very low carbohydrate" OR VLCKD OR "high fat diet") AND ("iron absorption" OR DMT1 OR ferroportin OR "iron deficiency") AND (obesity OR obese)	Language: English<br>Relevance threshold: ≥4.0/5.0		iron search; animal studies excluded at screening stage
**SciSpace TOTAL**	Four search strings combined; duplicates removed	Combined filters as above	**n = 325**	After combining 4 strings and removing within-database duplicates
**Combined Totals Across All Databases**				
**ALL DATABASES**	All search strings from PubMed, Google Scholar, and SciSpace combined; cross-database deduplication performed using title + author + year matching	Cross-database deduplication: 62 duplicates removed	**458 identified → 62 duplicates removed = 334 unique records**	PubMed (n=14) + Google Scholar (n=57) + SciSpace (n=325) + additional hand-searching (n=62 potential duplicates identified)

Screening Criteria Applied at Each Stage

Criterion	Type	Description	Applied at Stage	Threshold
**IC1**	**Inclusion**	Adults (≥18 years) classified as obese (BMI ≥30 kg/m^2) or with other validated clinical measures of obesity	Abstract + Full-text	≥4.0/5.0 (abstract); ≥4.5/5.0 (full-text)
**IC2**	**Inclusion**	Studies evaluating effects of Mediterranean diet, ketogenic diet, or intermittent fasting; caloric restriction or bariatric surgery studies providing mechanistic insights were also included	Abstract + Full-text	≥4.0/5.0 (abstract); ≥4.5/5.0 (full-text)
**IC3**	**Inclusion**	Quantitative data for at least one iron biomarker: serum iron, ferritin, transferrin saturation, hepcidin, hemoglobin, TIBC, or transferrin receptor	Abstract + Full-text	≥4.0/5.0 (abstract); ≥4.5/5.0 (full-text)
**EC1**	**Exclusion**	Preclinical and animal research (in vivo, in vitro, or computer simulations)	Abstract + Full-text	Automatic exclusion
**EC2**	**Exclusion**	Pediatric populations (age <18 years)	Abstract + Full-text	Automatic exclusion
**EC3**	**Exclusion**	Studies where the primary population was lean or overweight (BMI 25–29.9 kg/m^2) without stratified obese subgroup results	Abstract + Full-text	Automatic exclusion
**EC4**	**Exclusion**	Clinical studies that did not measure or report any indicator of iron status or deficiency	Full-text only	Automatic exclusion

Abbreviations: IC = Inclusion Criterion; EC = Exclusion Criterion; BMI = Body Mass Index; MeSH = Medical Subject Headings; TIBC = Total Iron-Binding Capacity; DMT1 = Divalent Metal Transporter 1; VLCKD = Very-Low-Calorie Ketogenic Diet; AI = Artificial Intelligence; EVOO = Extra-Virgin Olive Oil.

Search date: All databases searched through December 2025. No language restrictions applied at search stage; non-English articles excluded at screening stage. Hand-searching of reference lists of included systematic reviews and meta-analyses was performed to identify additional eligible studies.
